# Supplementary material for: Phylogenetic Analysis and Protein Modelling of Isoflavonoid Synthase Highlights Key Catalytic Sites towards Realising New Bioengineering Endeavours
Source: Bioengineering (Basel). 2022 Oct 24;9(11):609. doi: 10.3390/bioengineering9110609 (PMC9687675; doi:10.3390/bioengineering9110609)
Supplement: Supplementary file 1 [file bioengineering-09-00609-s001.zip › Sajid-et-al_Sup. Fingure.pptx]

## Slide 1
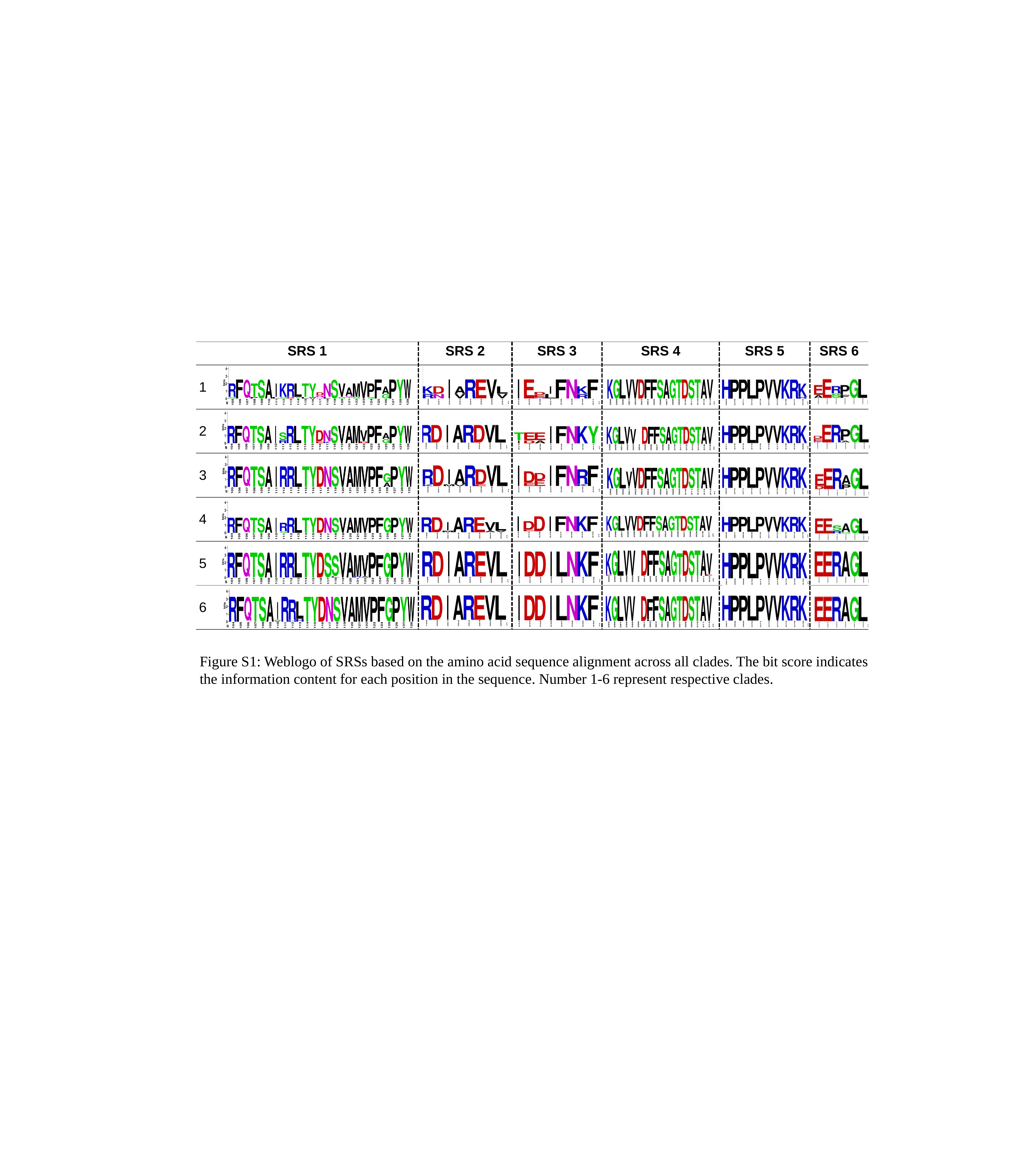

| SRS 1 | SRS 2 | SRS 3 | SRS 4 | SRS 5 | SRS 6 |
| --- | --- | --- | --- | --- | --- |
| 1 | | | | | |
| 2 | | | | | |
| 3 | | | | | |
| 4 | | | | | |
| 5 | | | | | |
| 6 | | | | | |
Figure S1: Weblogo of SRSs based on the amino acid sequence alignment across all clades. The bit score indicates the information content for each position in the sequence. Number 1-6 represent respective clades.

## Slide 2
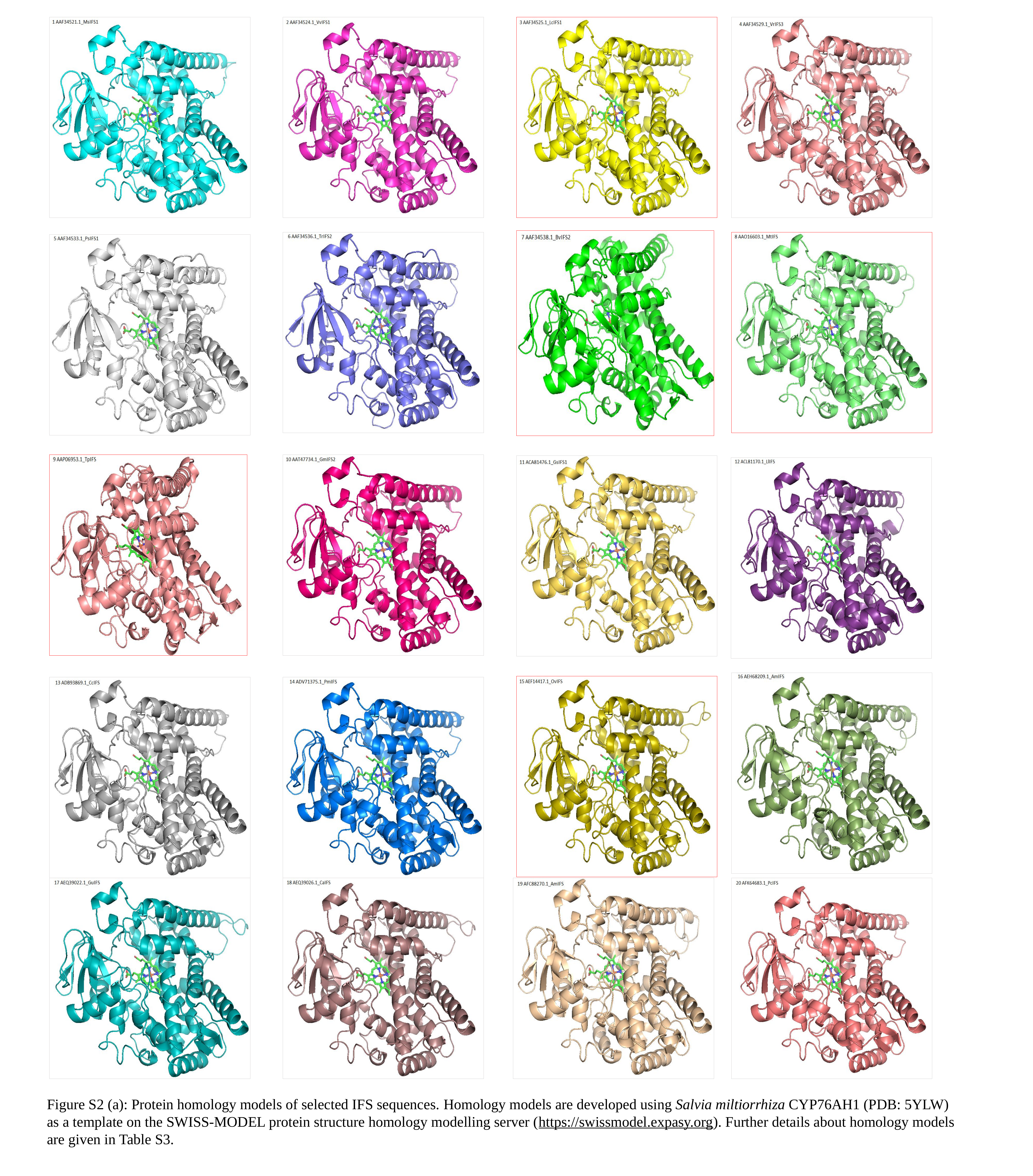

Figure S2 (a): Protein homology models of selected IFS sequences. Homology models are developed using Salvia miltiorrhiza CYP76AH1 (PDB: 5YLW) as a template on the SWISS-MODEL protein structure homology modelling server (https://swissmodel.expasy.org). Further details about homology models are given in Table S3.

## Slide 3
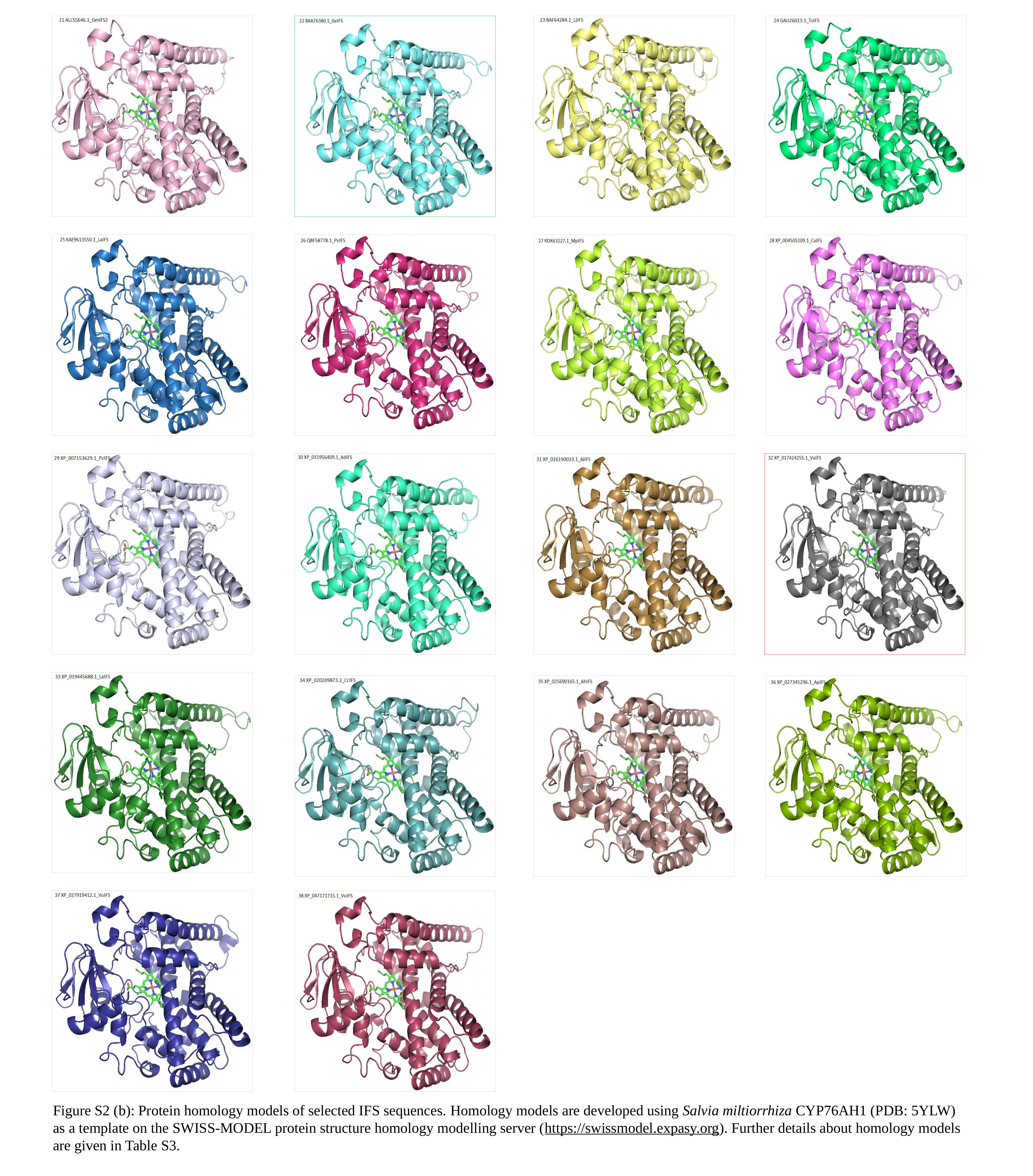

Figure S2 (b): Protein homology models of selected IFS sequences. Homology models are developed using Salvia miltiorrhiza CYP76AH1 (PDB: 5YLW) as a template on the SWISS-MODEL protein structure homology modelling server (https://swissmodel.expasy.org). Further details about homology models are given in Table S3.

## Slide 4
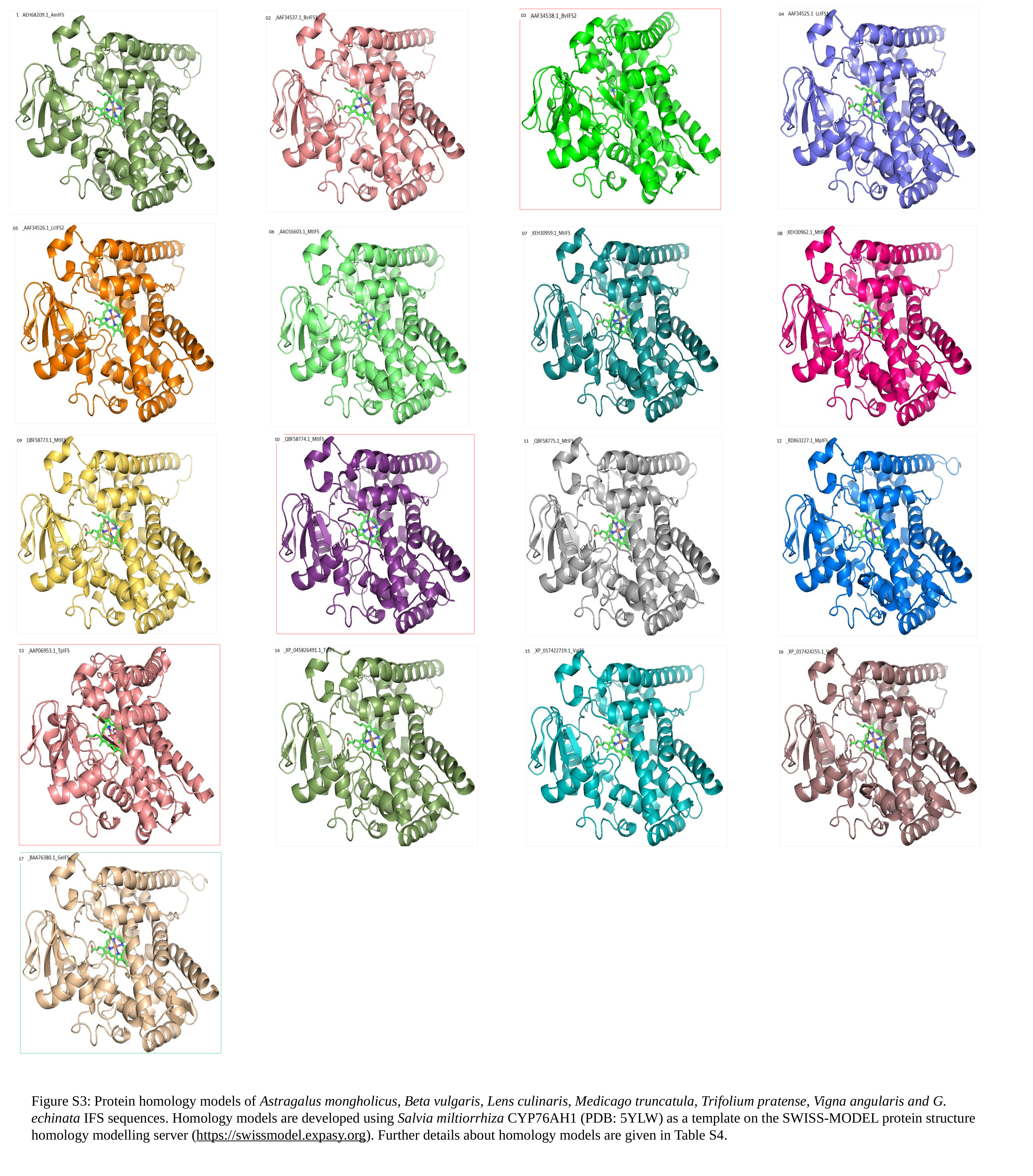

04
03
02
05
08
06
07
10
09
11
12
13
14
15
16
17
Figure S3: Protein homology models of Astragalus mongholicus, Beta vulgaris, Lens culinaris, Medicago truncatula, Trifolium pratense, Vigna angularis and G. echinata IFS sequences. Homology models are developed using Salvia miltiorrhiza CYP76AH1 (PDB: 5YLW) as a template on the SWISS-MODEL protein structure homology modelling server (https://swissmodel.expasy.org). Further details about homology models are given in Table S4.

## Slide 5
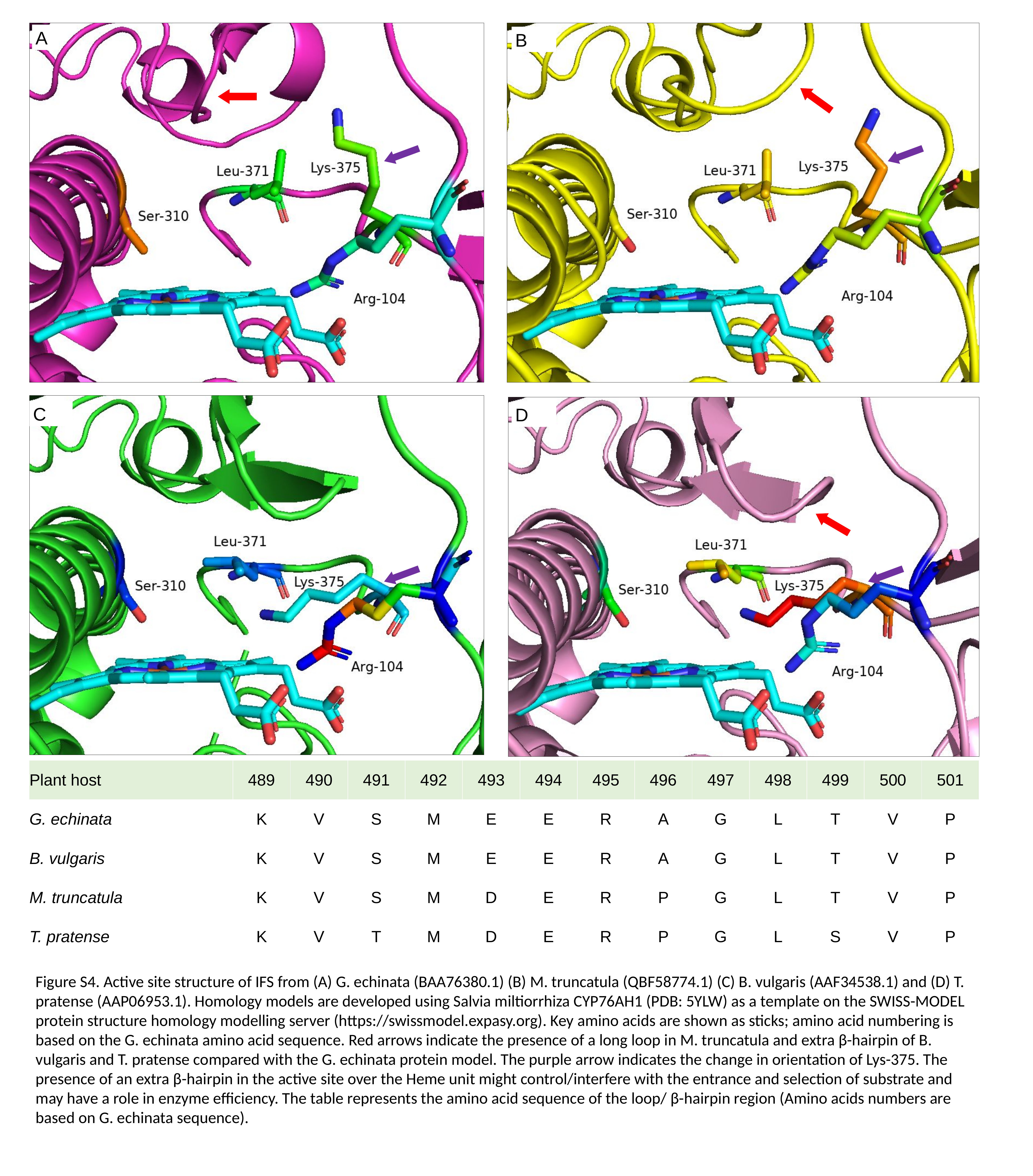

A
B
C
D
| Plant host | 489 | 490 | 491 | 492 | 493 | 494 | 495 | 496 | 497 | 498 | 499 | 500 | 501 |
| --- | --- | --- | --- | --- | --- | --- | --- | --- | --- | --- | --- | --- | --- |
| G. echinata | K | V | S | M | E | E | R | A | G | L | T | V | P |
| B. vulgaris | K | V | S | M | E | E | R | A | G | L | T | V | P |
| M. truncatula | K | V | S | M | D | E | R | P | G | L | T | V | P |
| T. pratense | K | V | T | M | D | E | R | P | G | L | S | V | P |
Figure S4. Active site structure of IFS from (A) G. echinata (BAA76380.1) (B) M. truncatula (QBF58774.1) (C) B. vulgaris (AAF34538.1) and (D) T. pratense (AAP06953.1). Homology models are developed using Salvia miltiorrhiza CYP76AH1 (PDB: 5YLW) as a template on the SWISS-MODEL protein structure homology modelling server (https://swissmodel.expasy.org). Key amino acids are shown as sticks; amino acid numbering is based on the G. echinata amino acid sequence. Red arrows indicate the presence of a long loop in M. truncatula and extra β-hairpin of B. vulgaris and T. pratense compared with the G. echinata protein model. The purple arrow indicates the change in orientation of Lys-375. The presence of an extra β-hairpin in the active site over the Heme unit might control/interfere with the entrance and selection of substrate and may have a role in enzyme efficiency. The table represents the amino acid sequence of the loop/ β-hairpin region (Amino acids numbers are based on G. echinata sequence).

## Slide 6
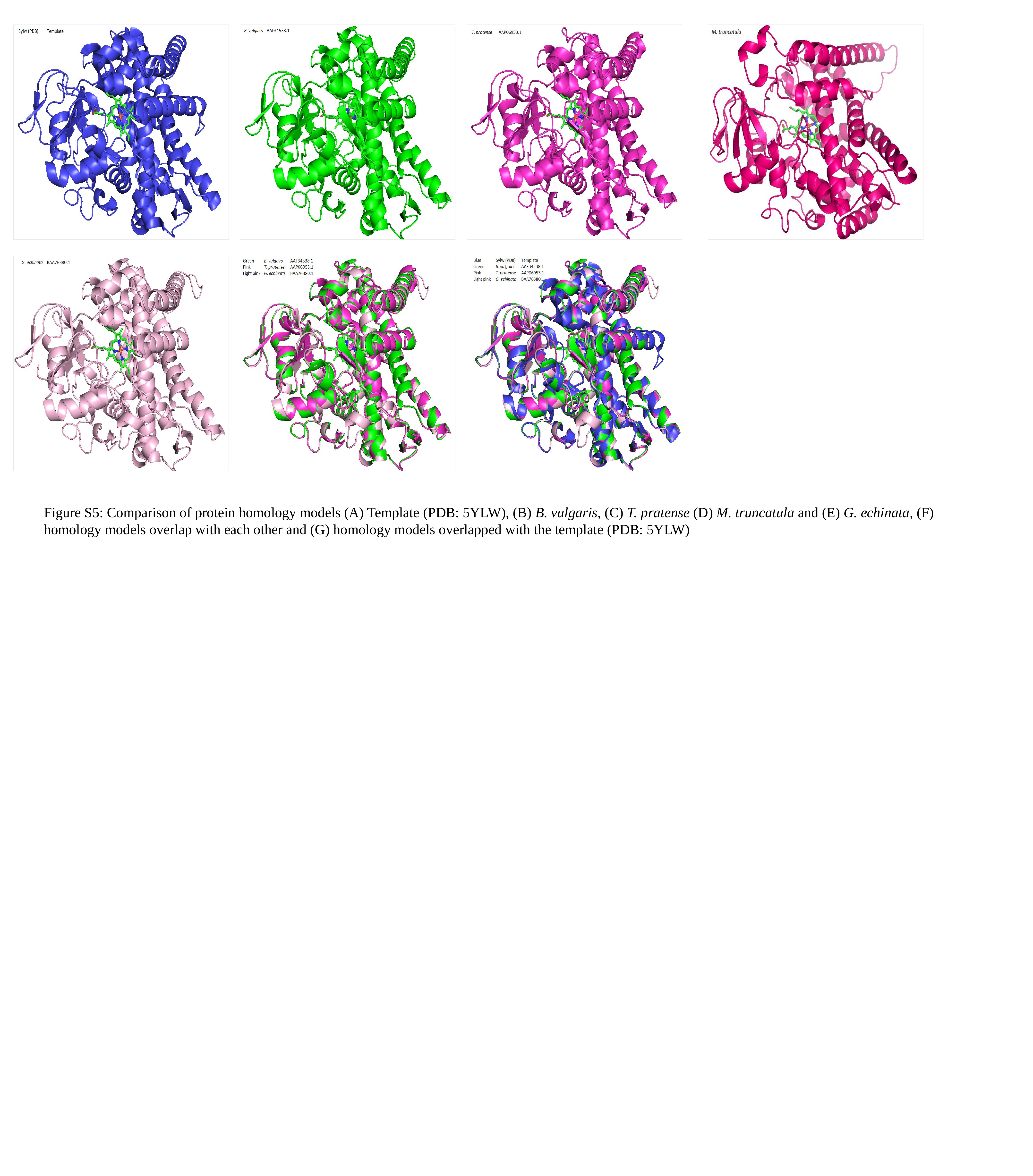

Figure S5: Comparison of protein homology models (A) Template (PDB: 5YLW), (B) B. vulgaris, (C) T. pratense (D) M. truncatula and (E) G. echinata, (F) homology models overlap with each other and (G) homology models overlapped with the template (PDB: 5YLW)

## Slide 7
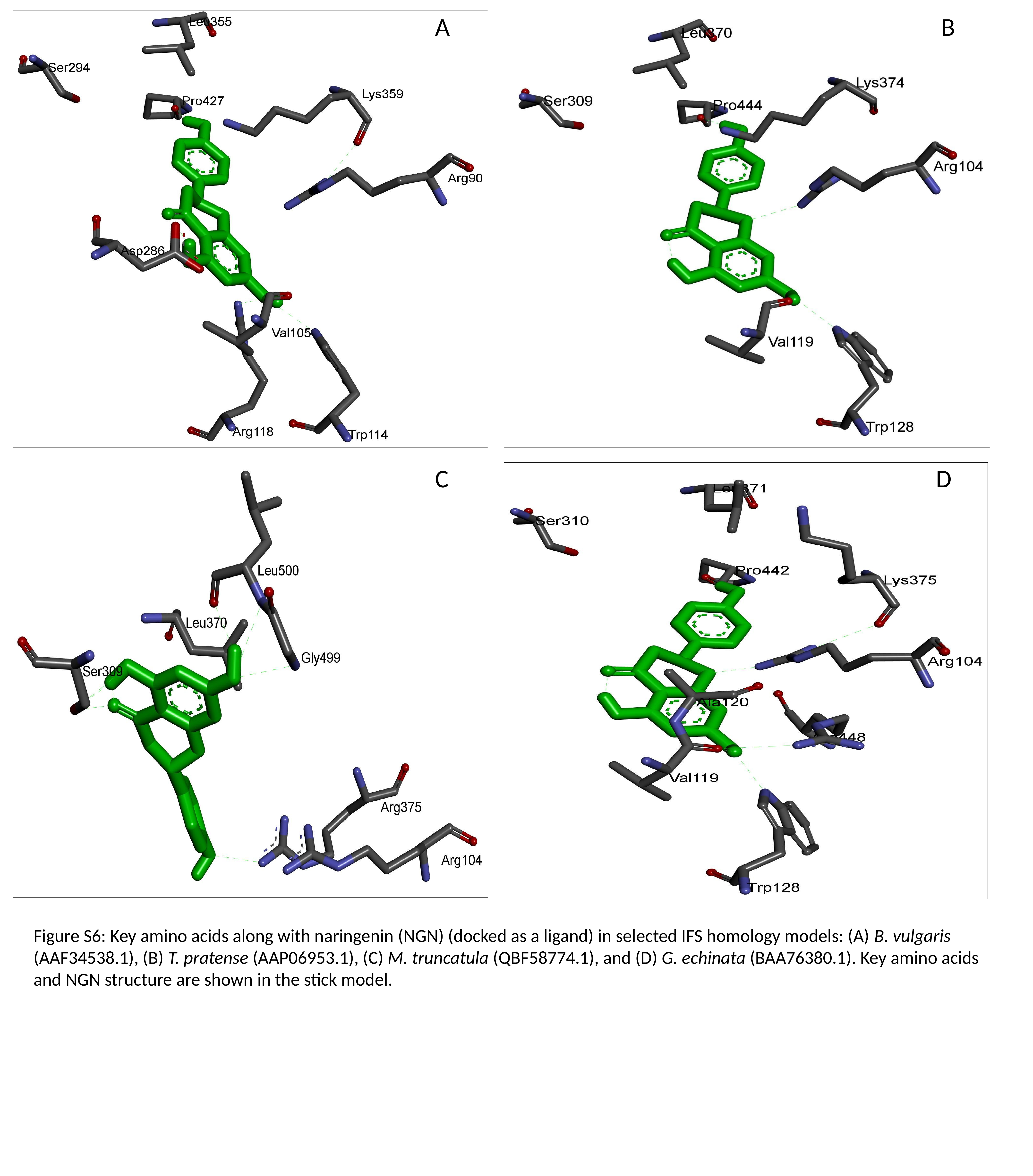

A
B
D
C
Figure S6: Key amino acids along with naringenin (NGN) (docked as a ligand) in selected IFS homology models: (A) B. vulgaris (AAF34538.1), (B) T. pratense (AAP06953.1), (C) M. truncatula (QBF58774.1), and (D) G. echinata (BAA76380.1). Key amino acids and NGN structure are shown in the stick model.
